# Early to sustained impacts of lethal radiation on circulating miRNAs in a minipig model

**DOI:** 10.1038/s41598-023-45250-9

**Published:** 2023-10-28

**Authors:** Nabarun Chakraborty, Gregory P. Holmes-Hampton, Aarti Gautam, Raina Kumar, Bernadette Hritzo, Betre Legesse, George Dimitrov, Sanchita P. Ghosh, Rasha Hammamieh

**Affiliations:** 1 Medical Readiness Systems Biology, CMPN, Walter Reed Army Institute of Research, Silver Spring, MD 20910 USA; 2 Armed Forces Radiobiology Research Institute, Uniformed Services University of the Health Sciences, Bethesda, MD 20889 USA; 3 The Geneva Foundation, US Army Center for Environmental Health Research, Fort Detrick, MD 21702-5010 USA

**Keywords:** Molecular biology, Physiology, Systems biology, Biomarkers, Signs and symptoms

## Abstract

Early diagnosis of lethal radiation is imperative since its intervention time windows are considerably short. Hence, ideal diagnostic candidates of radiation should be easily accessible, enable to inform about the stress history and objectively triage subjects in a time-efficient manner. Therefore, the small molecules such as metabolites and microRNAs (miRNAs) from plasma are legitimate biomarker candidate for lethal radiation. Our objectives were to comprehend the radiation-driven molecular pathogenesis and thereby determine biomarkers of translational potential. We investigated an established minipig model of LD70/45 total body irradiation (TBI). In this pilot study, plasma was collected pre-TBI and at multiple time points post-TBI. The majority of differentially expressed miRNAs and metabolites were perturbed immediately after TBI that potentially underlined the severity of its acute impact. The integrative network analysis of miRNA and metabolites showed a cohesive response; the early and consistent perturbations of networks were linked to cancer and the shift in musculoskeletal atrophy synchronized with the comorbidity-networks associated with inflammation and bioenergy synthesis. Subsequent comparative pipeline delivered 92 miRNAs, which demonstrated sequential homology between human and minipig, and potentially similar responses to lethal radiation across these two species. This panel promised to retrospectively inform the time since the radiation occurred; thereby could facilitate knowledge-driven interventions.

## Introduction

The need to build a comprehensive knowledge about the nuclear radiation exposure is more pertinent than ever given the permeation of ionizing radiation in our society’s framework. Not only has the threat of global nuclear events become more imminent in recent years^1^, but the escalated handling of nuclear radiation in industrial and medical sectors also pose risks for accidental radiation exposure. Exposure to ionizing radiation causes dose-dependent health effects and are characterized as Acute Radiation Syndrome (ARS). Adverse effects in humans can be induced after exposure to as little as 2 Gy^2,3^ of radiation resulting the damage to proliferating cells in the most radio-sensitive system, namely the hematopoietic ARS (H-ARS). H-ARS is characterized by depletion of circulating blood cells, induction of widespread inflammation and endothelial dysfunction^4–6^, which lead to potentially life-threatening infection and hemorrhaging^4,7^. Longitudinal and dose-dependent evaluation of H-ARS is imperative to develop precise intervention strategy.

Since, nearly all the radiation injured patients have preexisting confounders, such as cancer, animal models are possibly the best source to study the exclusive impacts of radiation. The in vivo models are crucial in the advancement of understanding the radiation effects as well as studying  the countermeasures and treatments for these effects^2^. Traditionally, the mouse models are the most popular in radiation research field due to its availability, cost, size, lifespan, and homology to human genotype and physiology^2,8,9^. However, significant differences between mouse and human in context of their skin textures and skin surface areas limit the applicability of mouse model in studying total body irradiation (TBI)^2^. The logistical burdens and mounting ethical concerns of using non-human primates in the radiation study somewhat outweighs its applicability to radiation research^2,9–12^.

Taking all these aspects in consideration, the Göttingen minipig (GMP) emerges as the most scientifically meaningful and logistically manageable animal model for advancing the knowledge of ARS pathophysiology. In general, the minipigs possess human-like immune system^13^, metabolism process^14^, and cardiopulmonary operation^15^. Swine are highly regarded as translation models for ischemic stroke, diabetes, obesity, traumatic brain injury, coronary artery disease, and infarction^13,15–21^. With a human-like proportionate ratios between body weight and skin area, comparable skin surface areas (0.5–1.0 m^2^ of minipig vs. 1.8–2.0 m^2^ in human), and similar skin textures, minipig emerges as a legitimate TBI model^22^. Furthermore, minipig genome scores 84% homology with human, which is higher than that of homology between mouse and human^23^. Recently, the U.S. Food and Drug Administration (FDA) approved the transplantation of porcine skin graft to humans and transplantation of heart valves from porcine origin has been common practice for the last 60 years. The close similarity between human and porcine anatomy and physiology, availability, and its extensive use in biomedical research make this model well-suited for biodosimetric applications and radiation countermeasure testing^2,9^.

Indeed, the minipig’s LD70/45 dose as used in the present study is lower than that in humans; nevertheless, previous studies characterized the GMP as a model of H-ARS showing that the GMP displays closely aligned ARS development and symptoms to those documented in non-human primates, canines, and humans^24–27^. Specifically, H-ARS in the GMP shows prodromal, latent, manifest, and morbidity or recovery phases of the disease as well as severe decline in peripheral blood cell counts, inflammation, hemorrhage, and organ dysfunction^24–27^. Further, the use of GMP H-ARS model have been characterized for countermeasure testing^26,28,29^. Tissue-specific patterns of radiation-induced gene expression changes have also been established in GMP H-ARS model^30^. In addition, we tested the miRNA sequence homology between human and minipig to foster confidence in our biomarker discover process.

Comprehensive assessment of radiation pathophysiology in this GMP model could inform the biomarker discovery for early diagnosis and retrospective triage of subjects exposed to TBI. Circulating microRNAs (miRNAs) are promising biomarkers of radiation damage^31–36^. Małachowska et al*.* reviewed the irradiation-inked miRNA profiles from 467 studies and suggested dose-dependent putative miRNA profiles for radiation exposure^31^. One of the miRNAs identified by Małachowska et al*.*^31^, miR-30c, which is associated with atherosclerosis and heart disease^31^, has been shown to play roles in radiation responses^35^ with countermeasure^37^. In a mouse ARS model, up-regulation of miR-27b, miR-126, and p53 target, miR-34a^34^ was found to be modulated by administration of a prophylactic radiation countermeasure, vitamin E isomer, gamma-tocotrienol. Of seven miRNAs that significantly changed at 24 h post-irradiation in rhesus macaques, miR-133b, miR-215, and miR-375 accurately distinguished exposure, and miR-30a and miR-126 accurately delineated based on survival outcome^33,38^. In their review, Singh and Pollard highlight the lack of miRNA studies in animal models outside of NHP and mice^33^. The field of miRNA research is still in its infancy and needs to be validated across multiple conditions^33^.

Changes in metabolomics following exposure to radiation is an emerging field^39^. Radiation-induced metabolic changes can be observed before the clinical onset of systems and accordingly could be used to augment current methods of triage for exposed individuals^39,40^. Metabolic profiles have been studied in mouse and rat biospecimens such as plasma urine, and tissues^41–45^; additionally previous reports demonstrated radiation-induced alterations of metabolic profiles^32,40^ in a mouse model of ARS. Metabolic profiles in radiotherapy patients have also been studied^46,47^, and increased levels of biomarkers involved in energy metabolism were observed in these patients^47^. A recent study in rhesus macaques has demonstrated that exposure to ionizing radiation leads to alterations to insulin response in tissues^48^. Additional studies have demonstrated alterations in amino acid distribution^49,50^ and levels of proteins involved in protein synthesis^51^ following exposure to ionizing radiation. As with miRNA profiling, the perturbation in metabolite landscape due to the ionizing radiation exposure is an understudied field.

In the current study, we aim to fill the gap using a GMP model of H-ARS, a pilot study with small sample size. We presented miRNA and metabolic profiling data from three animals that did not survive TBI at 2.2 Gy. This pilot LD70/45dose produced 70% lethality in the 45-day period post-TBI. To assess the temporal changes due to radiation, blood collected at various times post-irradiation with baselines established prior to TBI exposure. High throughput miRNA reads were screened to curate those with high sequence homology with human. We presented a novel algorithm to meet this objective. In addition, liquid chromatography-mass spectrometry (LC–MS) reads were annotated to find metabolites of interest. Since, no further validation of these spectral features were performed, these were essentially *putative* metabolites.

## Results

**Figure 1:**
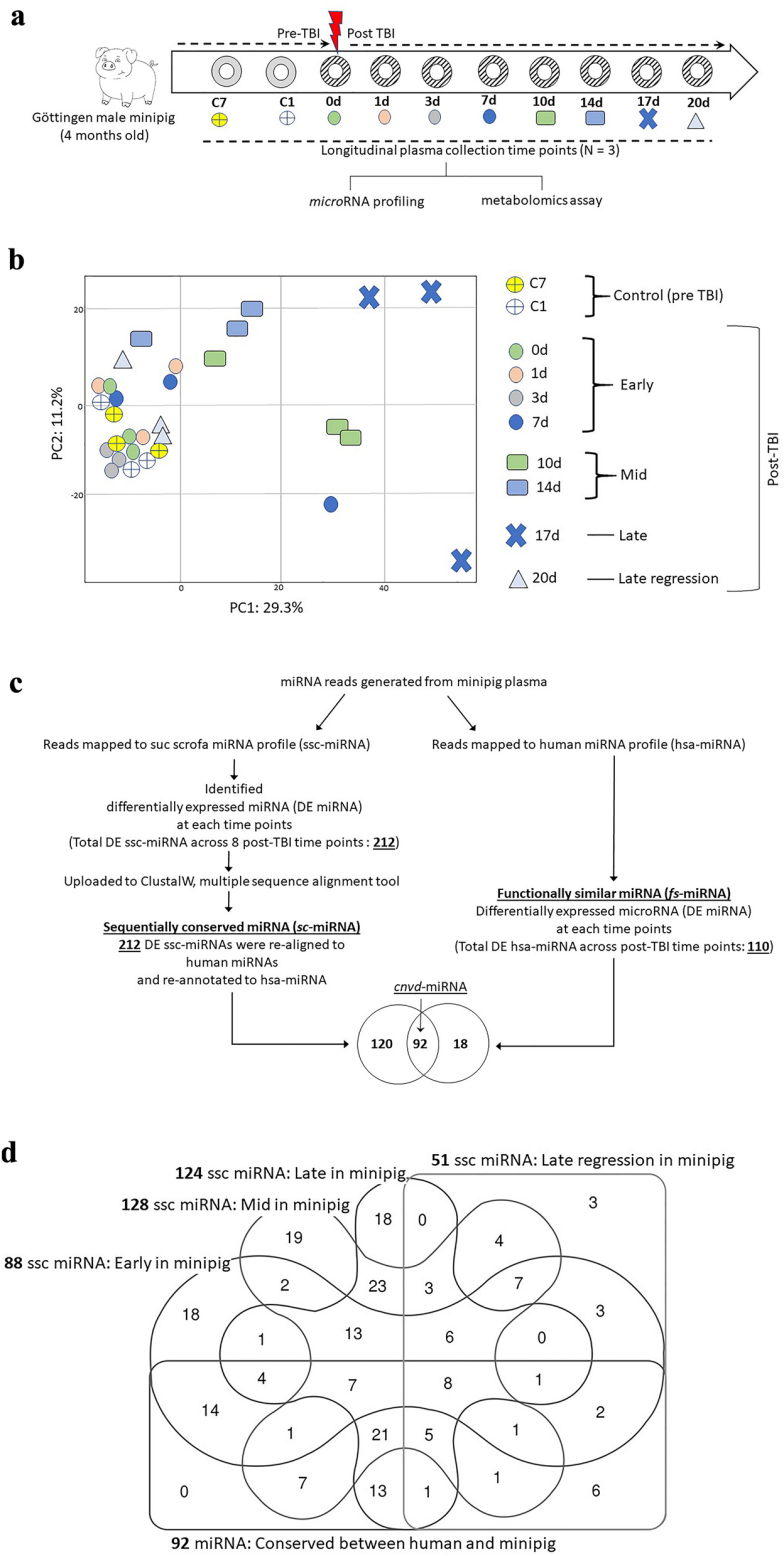
(**a**) The study design. The 4 months old male *sus surcofa* were exposed to lethal LD70/45 total body irradiation (TBI). The terminal plasma samples were collected at two time points before the irradiation, namely 7 days (C7) and 1 day (C1) pre-TBI. Six hours after TBI, the first terminal plasma samples were collected and it marked as 0d. Subsequently, terminal plasma samples were collected at seven time points, namely 1 day (1d), 3 day (3d), 7 day (7d), 10 day (10d), 14 day (14d), 17 day (17d) and 20 day (20d) post-TBI, respectively. Each time points were coded in the flow diagram and same codes were used in the next figure demonstrating the Principal Component Analysis (PCA). Plasma samples were used for microRNA (miRNA) and metabolite assays. (**b**) The PCA plot of all plasma miRNAs expressed in individual time points. PC1 and PC2 explained 29.3% and 11.2% of total variance, respectively. Seven days and one day pre-TBI time points were named as -7d and -1d, respectively and were labeled as wheel shapes colored by yellow and white, respectively. Post-TBI time points were divided into four phases based on their degree of separation in the Euclidian space from the pre-TBI samples. The early time points that essentially clustered together with baselines were named Early phase. This time-cluster included 0d, 1d, 3d and 7d post-TBI time points and were marked as the circles colored by green, orange, gray and blue, respectively. Ten day (10d) and 14d post-TBI time points were marginally separated from this baseline and Early phase conglomerate, which were named as Mid phase and labeled by squares colored by green and blue, respectively. Late phase or 17d post-TBI samples were clustered furthest from the conglomerate made by the baseline and Early phase. Blue colored ‘X’s labeled the Late phase. A light blue colored triangle marked the 20d post-TBI time points, which were named as Late Regression (LR) phase to acknowledge the fact that these samples clustered back to the conglomerate of baseline and Early phase. (**c**) A flow chart depicting an in silico model to identify the clinically actionable miRNAs. The miRNA reads were mapped to both sus sarcofa and human genome in parallel. The null hypothesis of this model was that these two independent read-mapping pipelines should generate a completely overlapping panel of miRNAs, since the sequences of miRNAs are believed to be conserved across the phylogenetic tree. Contrasting to the pre-TBI baseline, the differentially expressed miRNAs at individual post-TBI temporal phases were identified for these two parallel pipelines. The miRNA reads mapped to sus sarcofa delivered 212 ssc-miRNAs, which were differentially expressed in at least one of the four temporal phases, namely Early, Mid, Late and LR. Sequence alignment of these ssc-miRNAs to human genome delivered a re-annotated panel of 212 hsa-miRNAs. The parallel pipeline mapping the miRNA reads directly to human genome found 110 hsa-miRNAs, which were differentially expressed in at least one of the four temporal phases. There were 92 miRNAs (40% of combined list) conserved between this two sets of hsa-miRNAs. Figure S2 depicted all of these 92 miRNAs and Table 1 listed a subset of this list, which showed strong translational potential to be a putative panel of early markers of lethal radiation. (**d**) Venn diagram of ssc-miRNAs. The differentially expressed ssc-miRNAs of all four temporal phases were compared among themselves and to the 92 miRNAs that emerged conserved between the two parallel pipelines explained in Fig. 1c.

Four months old male GMPs were exposed to LD70/45 lethal dose of TBI. Baseline plasma samples were collected seven days (C7) and one day (C1) pre-TBI. First irradiated plasma samples were collected six hours after TBI and we called it 0d time point; subsequently plasma samples were drawn from the same three GMPs at 7 different post-TBI time-points, namely 1 day (1d), 3 day (3d), 7 day (7d), 10 day (10d), 14 day (14d), 17 day (17d) and 20 day (20d) post-TBI (Fig. 1a).

### Longitudinal expression dynamics of microRNA (miRNA)

The principal component analysis (PCA) plot revealed the underlying relationship among the time resolved miRNA expressions (Fig. 1b). The PC1 and PC2 explained 29.3% and 11.2% of total variance in the global miRNA landscape. Taking cues from the distributions of the samples across PCA plot, the time points were assigned ad hoc into four phases. Early phase included 0d, 1d, 3d and 7d post-TBI time points, which were marked by circles in Fig. 1b. Comprising 10d and 14d post-TBI time points, the Mid phase was marked by rectangles in Fig. 1b. Finally, the Late and LR phases included 17d and 20d post-TBI time points, respectively.

Differential analysis of Early phase found 45 upregulated and 43 down-regulated ssc-miRNAs from the pre-TBI baseline. Likewise, there were 22 upregulated and 106 down-regulated, 11 upregulated and 113 down-regulated, and 26 upregulated and 24 down-regulated ssc-miRNAs in Mid, Late and LR-phase, respectively. Together, there were 212 ssc-miRNAs, which were differentially expressed in at least one of the four temporal phases (Table S1A).

### Multiple sequence alignment between human and minipig miRNA assembly

Objective of the analysis pipeline was to determine a subset of these DE ssc-miRNAs that would have high translational potential (Fig. 1c). A multiple sequence alignments protocol^52^ identified those hsa-miRNAs, which were sequentially conserved to 212 DE ssc-miRNA; and we named these 212 ssc-miRNA as sequentially conserved-miRNA (*sc*-miRNA). In a parallel pipeline, the quality controlled NextSeq reads were *directly* blasted to human genome assembly and differential expression analysis was performed to curate 110 significantly different hsa-miRNAs from pre-TBI baseline. We labeled this second set as functionally similar miRNA (*fs*-miRNA). Ultimately, 212 *sc*-miRNAs and 110 fs-miRNAs were overlapped to find 92 miRNAs that were common between these two sets and named as conserved-miRNA (*cnvd*-miRNA). In the hierarchical clustering of these *cnvd*-miRNAs (Figure S1), four nodes were of interests, since they included those *cnvd*-miRNAs, which demonstrated longitudinally consistent trends of regulations (Fig. 1d and Table 1).

**Table 1:** A clinically actionable panel of microRNAs (miRNA) markers of early response to lethal total body irradiation (TBI).

*micro*RNA	Log_2_(fold change)				Biofunction
	Early	Mid	Late	LR	
miR-432-5p*	− 2.61	− 2.49	− 2.49	− 2.70	Inhibited expression is linked to pancreatic cancer^77^
miR-221-5p*	− 2.66	− 2.62	− 2.62	− 2.83	Inhibited expression promotes cancer^78^
miR-92b-5p*	− 2.75	− 2.62	− 2.62	− 2.83	Potential biomarker of heart failure^79^
miR-331-3p*	− 3.44	− 3.40	− 3.40	− 3.60	Linked to carcinogenesis^80^
miR-424-5p*	− 3.54	− 3.49	− 3.49	− 3.70	Identified as prognostic marker of multiple sclerosis^81^
miR-374b-5p*	− 4.05	− 4.00	− 4.00	− 4.21	Identified as prognostic marker of multiple sclerosis^81^
miR-376a-3p*	− 3.92	− 3.80	− 3.80	− 4.00	Linked to carcinogenesis^82^
miR-133a-3p*	− 4.44	− 3.10	− 9.61	–	Linked to carcinogenesis^80^
miR-545-3p*	− 4.31	− 4.19	− 4.19	–	Linked to cell migration, proliferation, and invasion^83^
miR-193a-5p	− 3.84	− 3.80	− 3.80	–	Inhibited expression linked to cancer^84^
miR-184*	− 3.80	− 3.76	− 3.76	–	Inhibited expression promotes cancer^85^
miR-542-5p	− 3.79	− 3.75	− 3.75	–	Inhibited expression promotes cancer^85^
miR-133b	− 2.57	− 2.44	− 2.44	–	Inhibited expression promotes cancer^85^
miR-218-5p*	4.02	3.27	–	4.83	Linked to carcinogenesis
miR-190b*	3.01	6.27	–	–	Promotes cancer
miR-1296-5p	− 2.53	− 2.40	–	–	Inhibited expression promotes cancer
miR-30c-1-3p	− 3.54	− 3.49	–	–	Inhibited expression promotes cancer

This miRNA panel met two selection criteria. (i) this is a subset of 92 cnvd-miRNAs, which are likely to be sequentially conserved and functionally similar miRNAs between human and minipig. (iii) In sus scrofa, these miRNAs were consistently expressed in at least two consecutive time points starting from the Early post-TBI phase. All of these miRNAs differentially expressed from the pre-TBI baseline with t-test *p* < 0.05; a subset of these miRNAs also met FDR cut-off 0.05, and those are marked with *.

### Longitudinal profile of miRNA enriched networks

**Figure 2:**
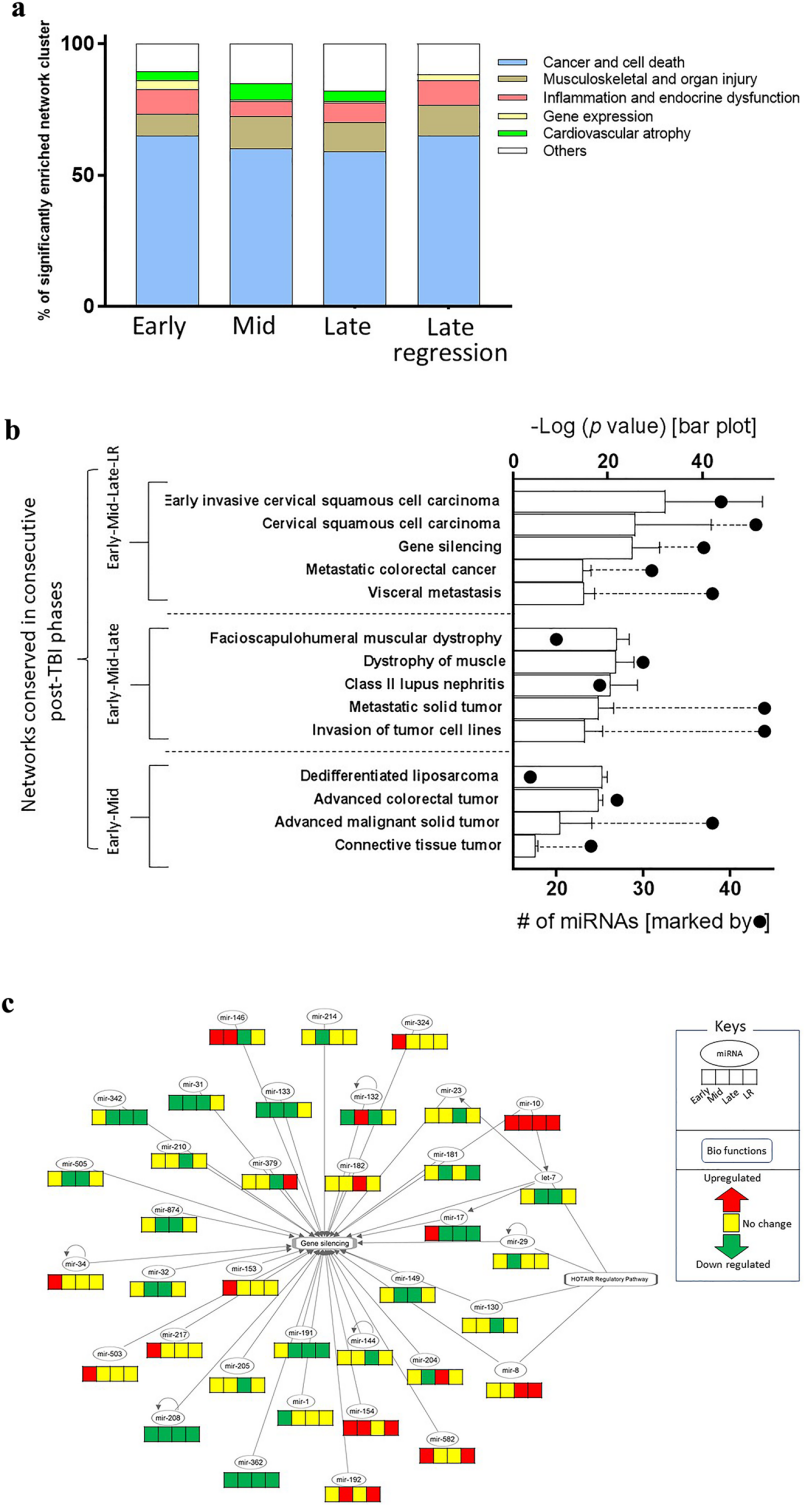
(**a**) Percentage share of the network categories across the temporal phases. The stacked bar plots represents the six dominating categories of networks. The group of networks labeled ‘others’ were linked to cell movement, cell cycle, neurological illness, developmental disorder etc. (**b**) Top five most significantly perturbed networks linked to Early time point. There were 37 networks that were consistently enriched across all four temporal phases, i.e. from Early to LR. Likewise, there were 41 and 4 networks that were consistently enriched in two other chronological combinations of temporal phases, i.e. from Early to Late and from Early to Mid, respectively. These networks were arranged in ascending order of their hypergeometric p values and five top ranked networks were selected from the two combinations of temporal phases, i.e. from Early to LR, and from Early to Late, respectively. All four networks from the third combination, i.e. from Early to Mid were selected. These networks emerged significantly enriched in at least two chronologically consecutive phases starting from the Early phase. The bar graphs were plotted against the top x-axis represents –log(*p*-value) and the solid black circles plotted against the bottom x-axis represents the number of miRNAs enriching individual networks. (**c**) Network linked to gene silencing. A regulatory network linked to the noncoding RNA residing in the homeobox C (HOXC) locus, termed HOTAIR emerged significantly altered within this framework of gene silencing network. In the network, the rectangular and oval nodes represent biofucntions and miRNAs, respectively. The four segments of the bar at the bottom of each miRNAs documented the regulations in four temporal phases arranged chronologically from left to right. The red, green and yellow colored segments represented up-, down and unchanged regulations in comparison to the pre-TBI baseline. The edges represent the relationship between two nodes; the arrow-headed edges represent activation and the straight-lined edges represent association between two nodes.

The entire list of 212 DE ssc-miRNAs enriched 206 networks, which were primarily sorted into six major categories, namely (i) cancer and cell death, (ii) musculoskeletal and organ injury, (iii) inflammation and endocrine dysfunction, (iv) gene expression, (v) cardiovascular atrophy and (vi) other; here the *other* set included cell movement, cell cycle, neurological illness, developmental disorder etc. A stacked bar chart (Fig. 2a) showed the longitudinal distribution of these network categories. The cancer related networks covered 60% of all networks across the timescale. There were 110 miRNAs enriching the cancer networks (Figure S3), of which four miRNAs, namely ssc-miR-374b-5p, ssc-miR-331-3p, ssc-miR-664-3p, ssc-mir-362 were consistently down-regulated and one miRNA, namely ssc-mir-10b was consistently up-regulated across the timescale. Furthermore, ssc-miR-374b-5p and ssc-miR-331-3p were among the *cnvd*-miRNA set. In addition to cancer network, other dominating network categories included the musculoskeletal and organ injury, and inflammation and endocrine dysfunction, and these two categories covered 10.8% and 7.9% of all networks.

Overall, there were 86, 178, 151 and 43 networks, which were most significantly perturbed in the Early, Mid, Late and LR phases, respectively (Table S2A). A Venn diagram (Figure S2) depicted the longitudinal distribution of these networks, as 39.8% of all these networks were perturbed starting from the Early phase. Ranked by the hypergeometric *p*-values, the most significantly perturbed networks were shown in Fig. 2b. Majority of these networks were linked to cancer. Figure S3 showed a hierarchical clustering of cancer related 106 ssc-miRNAs.

The gene-silencing network, a candidate of the gene expression category was significantly perturbed across the entire post-TBI time scale (Fig. 2c). The gene-silencing network was enriched by 36 ssc-miRNAs, of which ssc-miR-362 and ssc-miR-208 were consistently down-regulated and ssc-miR-10 were consistently upregulated across the four post-TBI phases. Furthermore, two networks were enlisted under musculoskeletal and organismal injury category, namely facioscapulohumeral muscular dystrophy and atrophy of muscle that emerged highly enriched across Early, Mid and Late phase. Muscular atrophy network was enriched by 36 miRNAs; notably, its two candidates, namely ssc-miR-374b-5p and miR-362 were consistently down regulated and ssc-miR-10 was consistently upregulated across the post-TBI phases.

### Longitudinal expression dynamics of putative metabolites

**Figure 3:**
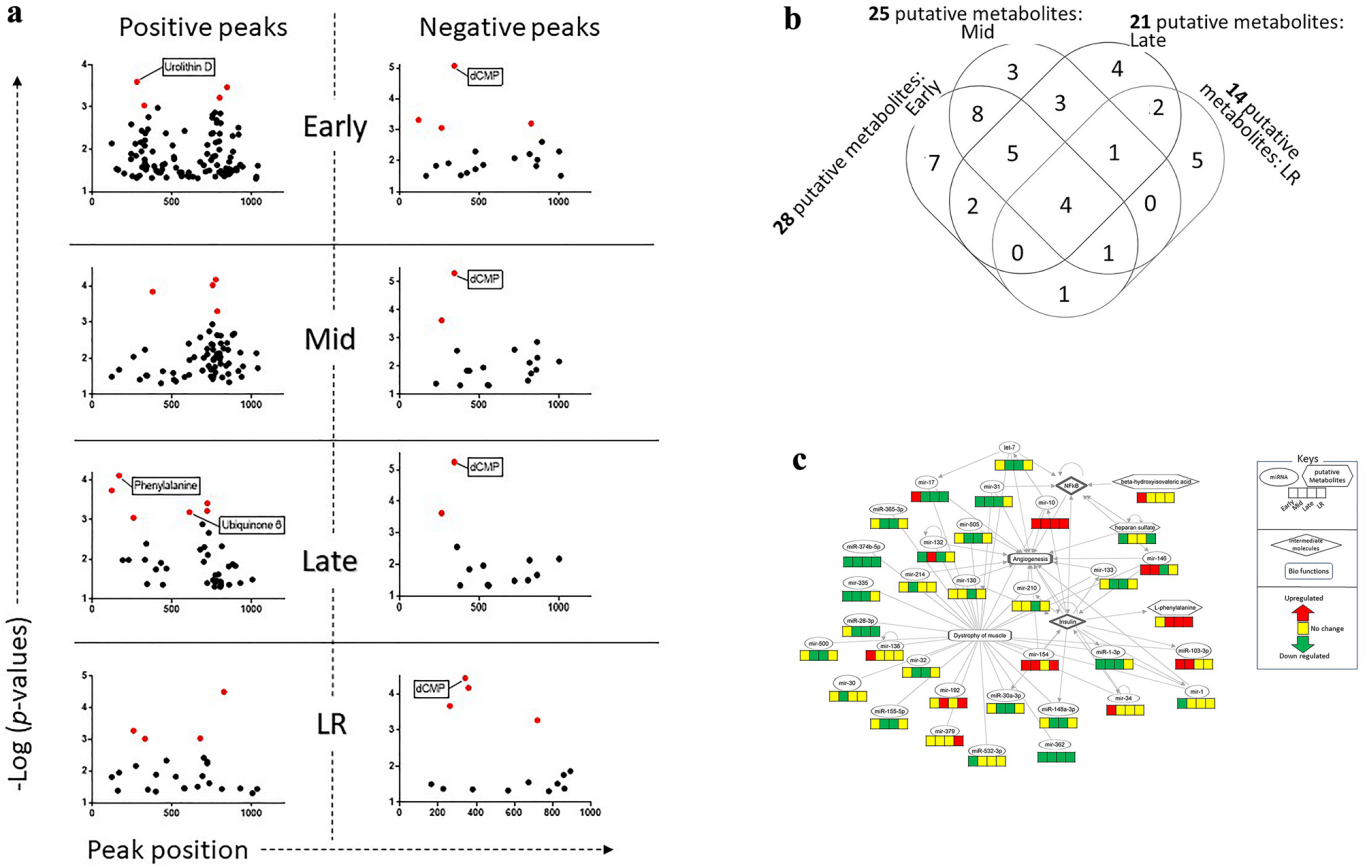
(**a**) Manhattan plot of differentially expressed (DE) spectral features. The left and right column of plots represented the positively and negatively charged peaks. Four temporal phases were arranged chronologically from top to bottom; the top row with two plots were linked to the Early phase and next three rows of plots were linked to Mid, Late and LR phases, respectively. The negative log transformed hypergeometric *p*-values of each spectral feature were presented in each plot, where the *p*-values measured the significance of divergence of the putative metabolites from the pre-TBI baseline. The red colored dots demonstrated highly significant spectral features [− log(p-value) > 3] and among these red dots, the HMDB-annotated spectral features were marked with their corresponding putative metabolite names. (**b**) Venn diagram of the Differentially abundant putative metabolites across the four temporal phases. The longitudinal distribution of these metabolites suggested that nearly 37% of them were consistently perturbed in at least two temporal phases starting from Early. These putative metabolites are listed in Table 2. (**c**) Network linked to dystrophy of muscle. This network was inferred from a group of DE miRNAs and differentially abundant putative metabolites. Angiogenesis emerged as one significant and pertinent network linked to muscular dystrophy. In the network, the rectangular, oval and hexagonal nodes represent biofucntions, miRNAs and putative metabolites, respectively. The rhombus shaped nodes were inserted to connect two molecules of interest, although the regulations of these molecules inside the rhombus nodes were not determined by present set of assays. The four segments of the bar at the bottom of each miRNAs and putative metabolites documented the regulations in four temporal phases arranged chronologically from left to right. The red-, green- and yellow-colored segments represented up-, down and unchanged regulations in comparison to the pre-TBI baseline. The edges represent the relationship between two nodes; the arrow-headed edges represent activation, and the straight-lined edges represent association between two nodes.

PCA plot of spectral features failed to show any trend. Table S3 listed all the spectral features across the time points. Further, we used the Manhattan plots to capture the impacts of TBI on metabolite profile. Here all the differentially abundant positively and negatively charged spectral features were shown with respect to their log transformed *p*-values (Fig. 3a). There were 106 positively charged and 19 negatively charged spectral features at Early phase. Likewise, there were 65, 38 and 26 positively charged and 18, 13 and 14 negatively charged spectral features in Mid, Late and LR phases. These spectral features were mapped to human metabolite database ID (HMDBID); and 29 (24 up-/4 down-regulated), 25 (23 up-/2 down-regulated), 21 (20 up-/1 down-regulated) and 13 (9 up-/4 down-regulated) spectral features were annotated by HMDB in the Early, Mid, Late and LR phases, respectively (Table S2B). More than 55% of all these HMDB-mapped putative metabolites emerged differentially expressed at the Early phase (Fig. 3b) and Table 2 listed a subset of the putative metabolites, which were showing consistent early regulations. These predictive panels of HMDBIDs (Table S1B) significantly enriched 10 functional networks; of which two networks, namely the binding of cell surface and the colony formation of hematopoietic progenitor cells emerged conserved between Early and LR phases (Table S2B). There were at least three networks, which were linked to the musculoskeletal atrophy, namely the catabolism of protein, hydrolysis of proteins and peroxidation of lipids; and the corresponding putative metabolites were seeded into the muscular atrophy networks along with the candidate miRNAs (Fig. 3c).

**Table 2:** A panel of putative metabolites with strong translational potential to be the marker of early response to lethal total body radiation (TBI).

HMDB ID	Name of putative metabolites	Biofunctions	Log_2_(fold change)			
			Early	Mid	Late	LR
Non-lipid metabolites/phosphates						
HMDB0001202	dCMP	Marker of oxidative damage and metabolic disorder caused by radiation	1.13	1.17	1.16	1.01
HMDB0011155	1-1z-alkenyl-2-acylglycerophosphate	An intermediate of ether lipid metabolism	0.35	0.41	0.38	0.51
Phospholipids and glycerophospholipid						
HMDB0009940	Phosphatidylinositol Phosphate(16:1/18:0)	Linked to cancer, cardiac atrophy, and nervous system disorder	0.20	0.24	0.18	0.18
HMDB0008951	Phophatidylethanolamine(16:1)	Linked to fatty acid metabolism and diabetes, often linked to radiation^86^	0.29	0.32	0.42	0.32
HMDB0007889	Phosphatidylcholine(36:4)	Linked to lipid metabolism	0.23	0.36	0.24	–
HMDB0009309	Phophatidylethanolamine(42:8)	Linked to lipid metabolism	0.34	0.33	0.33	–
HMDB0007881	Phosphatidylcholine(34:3)	Linked to carcinogenesis	0.39	0.35	0.26	–
HMDB0006588	Disialosyl galactosyl globoside	Linked to renal cell carcinoma	0.31	0.30	0.30	–
HMDB0007890	Phosphatidylcholine(36:5)	Linked to lipid metabolism	0.66	0.52	0.38	–
HMDB0012108	1-heptadecanoyl-2-hydroxy-sn-glycero-3-phosphocholine	Linked to lipid metabolism	0.60	0.50	–	–
HMDB0007963	Phosphatidylcholine(15:1)	Linked to lipid metabolism	0.73	0.51	–	–
HMDB0007883	Phosphatidylcholine(34:4)	Linked to lipid metabolism	0.39	0.47	–	–
HMDB0009573	Phophatidylethanolamine(44:8)	Linked to fatty acid metabolism and diabetes, often linked to radiation^86^	0.71	0.55	–	–
HMDB0007894	Phosphatidylcholine(38:1)	Linked to lipid metabolism	0.35	0.38	–	–
HMDB0007940	Phosphatidylcholine(33:2)	Linked to lipid metabolism	0.71	0.69	–	–
HMDB0007874	Phosphatidylcholine(32:2)	Linked to lipid metabolism	0.43	0.45	–	–
HMDB0007937	Phosphatidylcholine(33:0)	Linked to lipid metabolism	0.38	0.43	–	–

This putative metabolite panel met two selection criteria: (i) these putative metabolites are differentially expressed in sus scrofa from its pre-TBI baseline in at least one post-TBI phase; and (iii) in sus scrofa, these putative metabolites were consistently expressed in at least two consecutive time points starting from the Early post-TBI phase. The biofunctions, if not cited, were curated from https://hmdb.ca/metabolites. The putative list of metabolites were sorted into organic class of compounds, namely lipid and non-lipid metabolites, and all these spectral features were differentially expressed from the pre-TBI baseline with t-test *p* < 0.05.

## Discussion

### Model characteristics and study limitations

The prospect of circulating miRNAs as radiation biomarkers received additional traction by the growing number of studies that aimed to integrate miRNA and metabolite expressions in blood serum or plasma^32,53–55^. The integrative miRNA-metabolite readouts present dual benefits. Firstly, miRNA analysis can help in comprehending the stress history by exploiting its epigenetic characters^56^. Secondly, miRNA-metabolite integrative model is a great resource to predict the potential disease footprints, since together miRNAs and metabolites capture a wide range of biofunctional dynamics. The major challenge of finding radiation marker is the near unavailability of ‘pure’ clinical samples without confounding factors, such as cancer. Therefore, the appeal of studying large animal models in the context of TBI is enormous. Further, it is essential to apply in silico mechanism to find phylogenetically conserved markers of translational potential. We aim to bridge this knowledge gap in this pilot study by investigating a large animal model that was minipig, which share many important traits with human. Next, analytical pipeline curated those markers, which were likely to be sequentially homologues with human and functionally similar in responding to lethal TBI.

Present assays used GMPs’ plasma samples; no other biomatrix including serum was used here. Since, the expression values of these small molecules varies among different biomatrix, it is important to note that present results including the putative biomarkers are likely most applicable for plasma samples. In addition, it is beyond the scope of present study to find the cellular sources of the small molecules. This is a pilot study since the results are limited by a small sample size. Nevertheless, longitudinal analysis and multi-omics interrogation were undertaken to enhance statistical power.

### Highly adverse acute impact of total body irradiation

The immediate impact of lethal radiation was exhaustive to both miRNA and metabolite profile in the minipig model, and this condition essentially mirrored a previously published TBI mouse model^57^. Systematic differential expression analysis found that the largest portion of all DE miRNAs and metabolites were perturbed at the Early phase. PCA plot of miRNAs revealed an underlying longitudinal profile driven by host response to TBI. C1 and C2 samples were clustered with 0d, 1d, 3d and 7d; together these post-TBI time points were named as Early phase. Clustered separately from the Early phase in the PCA plot, two subsequent time points (10d and 14d post-TBI) were jointly named as Mid phase. Subsequent time point, 17d post-TBI diverged furthest from the pre-TBI, Early and Mid phases, respectively, and we binned 17d as the lone entrant of Late phase. Till the Late phase, the Euclidian divergence in PCA plot increased from the pre-TBI baseline in proportion to the times since irradiation, which mirrored the trend previously reported in mouse model^57^. However, this trend was breached at 20d post-TBI in GMPs, as this very late time point clustered back to the Early phase. Hence, we named 20d time point as LR phase. This incident potentially highlighted the differences between the mouse and minipig models. The proportionately larger animal model opened a scope to probe the effects of lethal radiation for a longer time window, which remained elusive to the mouse study^57^.

Interestingly, the longitudinal regression trend of miRNA expression profile was not mirrored by the network analysis. Majority of the networks were triggered at the Early phase underscoring the need of immediate intervention to combat lethal radiation. The cancer and cell death related networks were consistently perturbed across the post-TBI time course. Early inhibition of musculoskeletal fitness network, along with perturbed protein catabolism network suggested adverse impacts of radiation on musculoskeletal system and protein synthesis^58^. Working in concert, time-independent perturbations of metabolites were linked to cellular turnover, immunological onset^59^ and energy synthesis^60^. Early elevation of bioenergy related metabolites, such as dCMP and heparan sulfate was noteworthy and further substantiated the similar observations reported by the mouse TBI model^57^.

A large number of significantly altered putative metabolites were belong to the class of glycerophospholipids and this observation came to support recent studies that showed that a lipogenic environment could be created as a survival mechanism of carcinoma under radiotherapy^61^. In this context, it is important to note that several cancer promoting microRNAs were consistently downregulated and networks linked to cancer cell metastasis, tumorigeneses were perturbed from Early to late post-TBI.

### An in silico approach to determine cross-species conserved markers

A rather cohesive perturbations of miRNA-metabolite networks across different animal models^57,62^ essentially promoted these small molecules as putative radiation markers. However, there were multiple challenges to translate these candidates to clinically actionable items, since the heterogeneity between human and swine genomic makeups are more pronounced in the noncoding transcriptional elements than their coding regions^63^. Hence, we aimed to identify those miRNAs markers, which not only share high sequence homology between human and minipig, but also likely to present a cross-species conserved functional response to lethal radiation. Towards this objective, a multiple sequence alignment method curated the sequentially homologous miRNAs between minipig and human.

A parallel analysis curated those miRNAs, which could possibly exhibit functionally coherent response to TBI between human and minipig. In this in silico undertaking, the miRNA sequence reads were mapped to human miRNA assembly. Subsequent differential analysis mined a set of hsa-miRNAs, which were possibly captured a human-*like* TBI response. Using a Venn- based down selection approach, we curated 92 *cnvd*-miRNAs, which were common between these two sets, namely the sequentially conserved and functionally similar miRNAs between human and minipig, respectively. Hence, these markers should have high translational potential.

This list of 92 *cnvd*-miRNAs was an assortment of novel predictive candidates and acknowledged markers of radiation and cancer. For instance, miR-24-1-5p^64^, miR-30e-3p^65^, miR-28-5p^66^, miR-142-3p^67^, miR-148a-5p^68^, miR-455-3p^69^, miR-139-3p^70^, miR-1306-5p^71^ and several others mentioned in Table 1 were linked to various radiation and carcinogenesis studies. On the other hand, there are some candidates in the list of 92 *cnvd*-miRNAs, such as miR-30a-3p and miR-199b-5p, which had not previously linked to minipig radiation model.

We envision that subsets of 92 *cnvd*-miRNAs could be employed for various diagnostic purposes. For instance, a set of miRNAs including miR-432-5p, miR-221-5p, miR-92b-5p, miR-331-3p, miR-424-5p, miR-376a-3p and miR-374b-5p were consistently down regulated across entire time elapsed since irradiation. This minipig-specific time span is equivalent to approximately 0–4 months up till nearly 12 months of human life^72^ post-TBI. Hence, these miRNAs could be assayed as time-independent irradiation markers.

Likewise, a subset of miRNAs in 92 *cnvd*-miRNAs were exclusively expressed immediately after irradiation, which is equivalent to nearly 0–4 months of human life^72^. For instance, following miRNAs were exclusively upregulated immediately post-TBI: miR-218-5p, miR-96-5p, miR-671-5p, miR-424-3p, miR-490-3p, miR-296-5p, miR-769-3p, miR-27b-5p, miR-190b, miR-374b-3p, miR-193a-3p, miR-202-3p, miR-885-3p and miR-217. On the other hand, following set was exclusively down regulated immediately post-TBI (note, this list precludes those, which were already mentioned under consistent marker list): miR-532-3p, miR-885-5p, miR-545-5p, miR-1296-5p, miR-133b, miR-206, miR-30c-1-3p, miR-676-3p, miR-542-3p, miR-542-5p, miR-184, miR-193a-5p, miR-139-5p, miR-545-3p, miR-133a-3p, miR-133a-5p and miR-1306-3p.

Together, the time-independent and early-TBI markers can be greatly beneficial in immediately determine any incident of irradiation assault. Table 1 has a refined set of markers of high translation potential. Acute effects of radiation are generally asymptomatic; indeed, the early symptoms are easy to be misdiagnosed with regular diarrhea, metabolic dysfunction etc. Standard Geiger Counter device often gives false-low reading after being saturated by high radiation dose^73^. Hence, an objective detection of lethal dose of radiation at early time points is one of the major present capability gaps^73^, which could be addressed by the abovementioned subsets of 92 *cnvd*-miRNAs.

Further capability gap includes the lack of any method to inform the time elapsed since the radiation exposure. Such knowledge will be highly useful to the clinicians because the impact of radiation on host’s health is time sensitive. We envision that certain subsets of 92 *cnvd*-miRNAs, such as those, which were exclusively regulated at the intermediate (e.g., equivalent to 5–9 months of human life post radiation) or late (e.g., approximately 12 months of human life post-irradiation) could be used to retrospectively triage the subjects based on time since the irradiation exposure.

## Conclusions

System Biology integration of circulating small molecules in plasma suggested adverse effects of lethal radiation that triggered multiple cancer network and developed a pro-carcinoma environment. Utilizing a novel analytical pipeline, we presented a putative panel of biomarkers that showed sequence homology with human and likely to respond to TBI in a phylogenetically coherent manner. An extensive citation search also fostered confidence about the relevance of several candidates to radiation. Systematic investigation of these markers would yield time sensitive miRNA-metabolite panel capable of revealing radiation history, triage subjects based on the time since radiation exposure and predicting disease onset.

## Materials and methods

### Ethics statement

All procedures pertaining to animals were reviewed and approved by the Armed Forces Radiobiology Research Institute (AFRRI) Institutional Animal Care and Use Committee (IACUC) using the principles outlined in the National Research Council’s Guide for the Care and Use of Laboratory Animals and performed in accordance with relevant guidelines and regulations. Animal studies were conducted in compliance with ARRIVE (Animal Research: Reporting of In Vivo Experiments) guidelines.

### Animal husbandry

Three male Göttingen minipigs* (Sus scrofa domestica)* were acquired from Marshall BioResources (North Rose, NY) between ages 3–5 months and weighing 8.5–10.5 kg. GMPs were individually housed (with social contact maintained through slatted cages) in the AFRRI vivarium on arrival and were quarantined and acclimated for two weeks. Water was provided ad libitum and twice daily meal portions (Mini-Swine Diet 8753, Envigo Teklad Diets, Madison, WI) were determined by supplier instruction according to animal weight.

### Irradiation and dosimetry

Following the quarantine period, GMPs were exposed to 2.2 Gy ^60^Cobalt (^60^Co) TBI (dose rate ~ 0.6 Gy/minute; unilateral sequential raising of the two ^60^Co sources) in the AFRRI Cobalt Radiation Facility. Dosimetry was confirmed as described previously^30^. Anesthesia for irradiation was as follows: 2–5% isoflurane supplied by mask (5% for induction; 2% for maintenance) followed by IM injection of TelazolVR (Zoetis Pharmaceuticals, Salisbury, MD; 100 mg/mL, 2 mg/kg) and Xylazine (Aspen Veterinary Resources Ltd; Loveland, CO; 50 mg/mL, 1 mg/kg). Post-irradiation, GMPs were monitored closely until fully ambulatory.

### Blood collection

Animals were anesthetized with 5–2% isoflurane (as described above) and blood was collected from peripheral veins into anticoagulated tubes on the day of irradiation (day 0) and days 1, 3, 7, 10, 14, 17, and 20 following exposure (Fig. 1a). Baseline values were established in blood collected 7 and 1 days prior to irradiation. The time points were categorized into four phases, Early (days 0, 1, 3 and 7), Mid (days 10 and 14), Late (day 17), and LR (day 20) of H-ARS. Complete blood counts were determined in whole blood; plasma was separated by centrifugation and stored at -80˚C until use.

### Treatment, monitoring, and supportive care

Days 1 and 8 post-TBI, GMPs received 5% dextrose subcutaneously (animals comprised control group of a countermeasure testing study^29^; injection volume was equivalent to volume of drug injected). Indicators of animal health (temperature, activity, posture, stool, vomit, respiratory activity and rate, and anorexia) were monitored twice daily. At time of blood collections, heart rate, lung sounds, perfusion, hydration, and body weight were assessed. Supportive care (prophylactic ciprofloxacin and amoxicillin/clavulanate (p.o.)) was administered when absolute neutrophil counts were < 500/μL as described previously^26^.

### Euthanasia

During daily health checks, GMPs were also monitored for pre-determined criteria that necessitated unscheduled euthanasia: detection of one absolute criteria (non-responsiveness, dyspnea, or hypothermia) or four or more non-absolute criteria (hyperthermia, anorexia, anemia, vomiting/diarrhea, lethargy, vestibular signs, prolonged hemorrhage (bruising, petechiae, frank bleeding)). Anesthetized animals (2–5% isoflurane and Xylazine and Telazol IM injection (as described above)) were humanely euthanized with an IV injection of Euthasol® (sodium pentobarbital; Covertus, Elizabethtown, PA; 1 mL/4.5 kg).

### miRNA sequencing analysis

Five microliter (µL) of GMP plasma sample ligated with adapters to construct the sequencing libraries using the TruSeq small RNA Sample Preparation kit and a multiplexed pool consisting of equimolar amounts of small RNA-derived libraries was size selected. Illumina NextSeq platform was used to generate 5 million reads for miRNA profiling^57^. Preprocessing of raw base calls and sample de-multiplexing were performed using the standard open-source tool bcl2fastq2 v2.20 (Illumina Inc., San Diego, CA, USA). Reads were quality filtered, adaptor trimmed in silico using Cutadapt software v1.16, and mapped against *Sus scrofa* v5 (ssc5) reference genome assembly using Bowtie v2.2.3.0 aligner. Next, these mapped reads were re-mapped to the established *Sus scrofa* miRNAs (ssc-miRNA) that was archived in miRBase. Using miRDeep2 core algorithm, we conducted ssc-miRNA detection and de novo ssc-miRNA prediction from ss5 reference genome assembly of minipig. The known ssc-miRNA quantification was determined using miRDeep2 quantification function. Count data was further analyzed using edgeR v3.6 downloaded from R Bioconductor package. ssc-miRNA reads less than 5 read counts (cpm > 0.5) in 10% of sample population were filtered out and the remaining counts were normalized by TMM normalization method after library size factor calculations. Sample library size distribution and per sample ssc-miRNA read counts matrix were generated to see overall quality of the data and PCA of the ssc-miRNAs was conducted to uncover longitudinal impact of TBI (Fig. 1b). The data file is uploaded at the Gene Expression Omnibus publicly available repository (GEO, https://www.ncbi.nlm.nih.gov/geo/info/linking.html); the accession number is GSE244507.

Differential expression (DE) analysis was performed using edgeR, where data was fit under negative binomial generalized log-linear model to the read counts for each miRNA. The baseline was the pool of three pre-TBI time points, and the gene pair-wise analysis identified DE ssc-miRNAs at each time points, at the cut-off moderate t-test *p* < 0.05.

Subsequent analysis was focused on curating those DE ssc-miRNAs, which would be sequentially conserved and functionally similar between human and minipig exposed to TBI (Fig. 1c). Briefly, we pooled all of these DE ssc-miRNAs to seed them into a multiple sequence alignment tool, namely ClustlW^52^. Here a pairwise sequence alignment between DE ssc-miRNAs and human reference genome assembly (version hg38) was conducted with specified loci position to meet the following parameters, namely the k-tuple word size 1 and window size 5 with top diagonals of 5 with percent method. Gap penalty of 10 and penalty of extension 0.1 with BLOSUM scoring weight matrix were applied. As a result, we identified a set of ssc-miRNAs, which were sequentially homologues to the known human miRNAs. We named this set as sequentially conserved-miRNA or *sc*-miRNA.

In parallel, we used the miRBase to align the quality filtered and adaptor-trimmed reads to the human genome assembly, hg38. Thereby, we followed the previously described analysis pipeline with one important difference, namely the miRDeep2 core algorithm in miRBase curated known hsa-miRNAs, not ssc-miRNA. The resultant hsa-miRNA matrix was quality filtered and the counts were normalized to generate per sample hsa-miRNA set. Differential expression analysis performed at each of the eight post-TBI time points found those hsa-miRNAs, which were significantly different from the baseline formed by the pool of three pre-TBI data, with a cutoff of moderate t-test *p* < 0.05. We named this set of hsa-miRNA as functionally similar-miRNA or *fs*-miRNA. Subsequent Venn approach identified 92 miRNAs that were conserved between *sc*-miRNA and *fs*-miRNA.

### Metabolomics assay

The untargeted metabolomics assay were performed following the previously published protocol^32,74^. Before sample preparation, the sample sequence was randomized to avoid bias. For the metabolomics assay preparation, 75 µL of an extraction solution containing internal standards made up of 2.5 mL water, 3.5 mL methanol, 4.0 mL isopropanol, 10 µL debrisoquine (1 mg/mL in ddH2O), and 50 µL of 4-nitrobenzoic acid (1 mg/mL in Methanol) (per 10 mL) was added to a 25 µL aliquot of plasma. The samples were vortexed and incubated on ice for 20 min. Next, 100 µL of chilled acetonitrile was added, the samples were vortexed, then were incubated at − 20 °C for 15 min. Lastly, the samples were centrifuged at 4 °C and the supernatant was transferred to a MS vial for LC–MS analysis.

A volume of 2 µL of each prepared sample was injected onto a Waters Acquity BEH C18 1.7 μm, 2.1 × 50 mm column for metabolomics and a Waters Acquity CSH C18 1.7 μm, 2.1 × 100 mm column for lipidomics using an Acquity UPLC system coupled to a Xevo G2-S quadrupole-time-of-flight mass spectrometer with an electrospray ionization source (UPLC-ESI-QToF-MS) (Waters Corporation, Milford, MA). The mobile phases consisted of 100% water (solvent A), 100% acetonitrile containing 0.1% formic acid (solvent B), and 100% isopropanol with 0.1% formic acid (solvent C). Each of the solvents contained 0.1% formic acid and solvent A for the lipidomics acquisition contained an additional 10 mM ammonium formate.

The solvent flow rate for the metabolomics acquisition was set to 0.5 mL/min with the column set at 60 °C. The LC gradient was as follows: Initial—98% A, 2% B; 0.5 min—98% A, 2% B; 4.0 min—40% A, 60% B; 8.0 min—2% A, 98% B; 9.0 min—2% A, 98% B; 9.5 min—11.8% B, 88.2% C; 11.0 min—11.8% B, 88.2% C; 11.5 min—50% A, 50% B; 12.0 min—98% A, 2% B; 13.0 min—98% A, 2% B.

The column eluent was introduced into the Xevo G2-S mass spectrometer by electrospray operating in either negative or positive electrospray ionization mode. Positive mode had a capillary voltage of 3.00 kV and a sampling cone voltage of 30 V. Negative mode had a capillary voltage of 2.00 kV and had a sampling cone voltage of 30 V. The desolvation gas flow was set to 600 L/hour and the desolvation temperature was set to 500 °C. The cone gas flow was 25 L/hour and the source temperature was set to 100 °C. The data were acquired in the sensitivity MS mode with a scan time of 0.300 s and an interscan time of 0.014 s. Accurate mass was maintained by infusing Leucine Enkephalin (556.2771 [M + H]^+^/554.2615 [M − H]^−^) in 50% aqueous acetonitrile (2.0 ng/mL) at a rate of 10 µL/min via the Lockspray interface every 10 s. The data were acquired in centroid mode with a 50.0 to 1200.0 m/z mass range for Time-of-flight mass spectrometry (TOF-MS) scanning. An aliquot of each sample was pooled and used as a quality control (QC) which represented all metabolites present. This QC sample was run at the beginning of the sequence to condition the column and then injected every 10 samples to check mass accuracy, ensure presence of internal standard, and to monitor shifts in retention time and signal intensities.

The untargeted data acquired were first converted to the NetCDF unified data format using the Databridge tool in MassLynx (Waters Corporation, Milford, MA). An in-house implementation of the XCMS R package (Scripps Institute, La Jolla, CA) was used for spectral features detection with ordered bijective interpolated warping algorithm utilized for retention time correction and parameters optimized using the Isotopologue Parameter Optimization (IPO) R package^75^. The mass to charge ratio and retention time (mzrt) features were normalized based on the internal standards (debrisoquine and 4-nitrobenzoic acid present in the extraction solution in positive and negative modes respectively) as well as QC-RLSC (QC robust LOESS signal correction) normalization. The differential expression analysis curated those time point-specific spectral features that were altered from the pre-TBI baseline with a cutoff of moderate t-test *p* < 0.05. The differentially expressed spectral features were annotated using CEU Mass Mediator database (www.ceumass.eps.uspceu.es) and the molecules were screened based on the following guideline: (a) Ppm error less than 1; (b) chemical formula comprised of the adducts: + H, − H, + Na, + K, + NH4, -Cl and (c) chemicals belonged to the type of endogenous mammalian^57,76^.

### Statistical analysis

All data generated or analyzed during this study are included in this published article and its supplementary information files.

GeneSpring v.13 (Agilent Technologies, Inc., Santa Clara, CA) was used to find statistically significant markers, compute principal component (PC) analysis and clustering analysis. Hierarchical clustering was conducted using Similarity Measure: Squared Euclidean similarity measures and Wards linkage rule. Prism v.8 (GraphPad, Inc. San Diego, CA) was used for data visualization.

Ingenuity pathway analysis v. 21.0 (Agilent, Inc.) was used for functional analysis.

### Supplementary Information

Supplementary Figures.

Supplementary Tables.

## Data Availability

All data generated or analyzed during this study are included in this published article and its supplementary information files. Raw data have been uploaded to the Gene Expression Omnibus publicly available repository (GEO, https://www.ncbi.nlm.nih.gov/geo/info/linking.html); the accession number is GSE244507.
