# Supplementary material for: Early to sustained impacts of lethal radiation on circulating miRNAs in a minipig model
Source: Sci Rep. 2023 Oct 28;13:18496. doi: 10.1038/s41598-023-45250-9 (PMC10613244; doi:10.1038/s41598-023-45250-9)
Supplement: Supplementary file 1 — Supplementary Figures. [file 41598_2023_45250_MOESM1_ESM.pptx]

## Slide 1
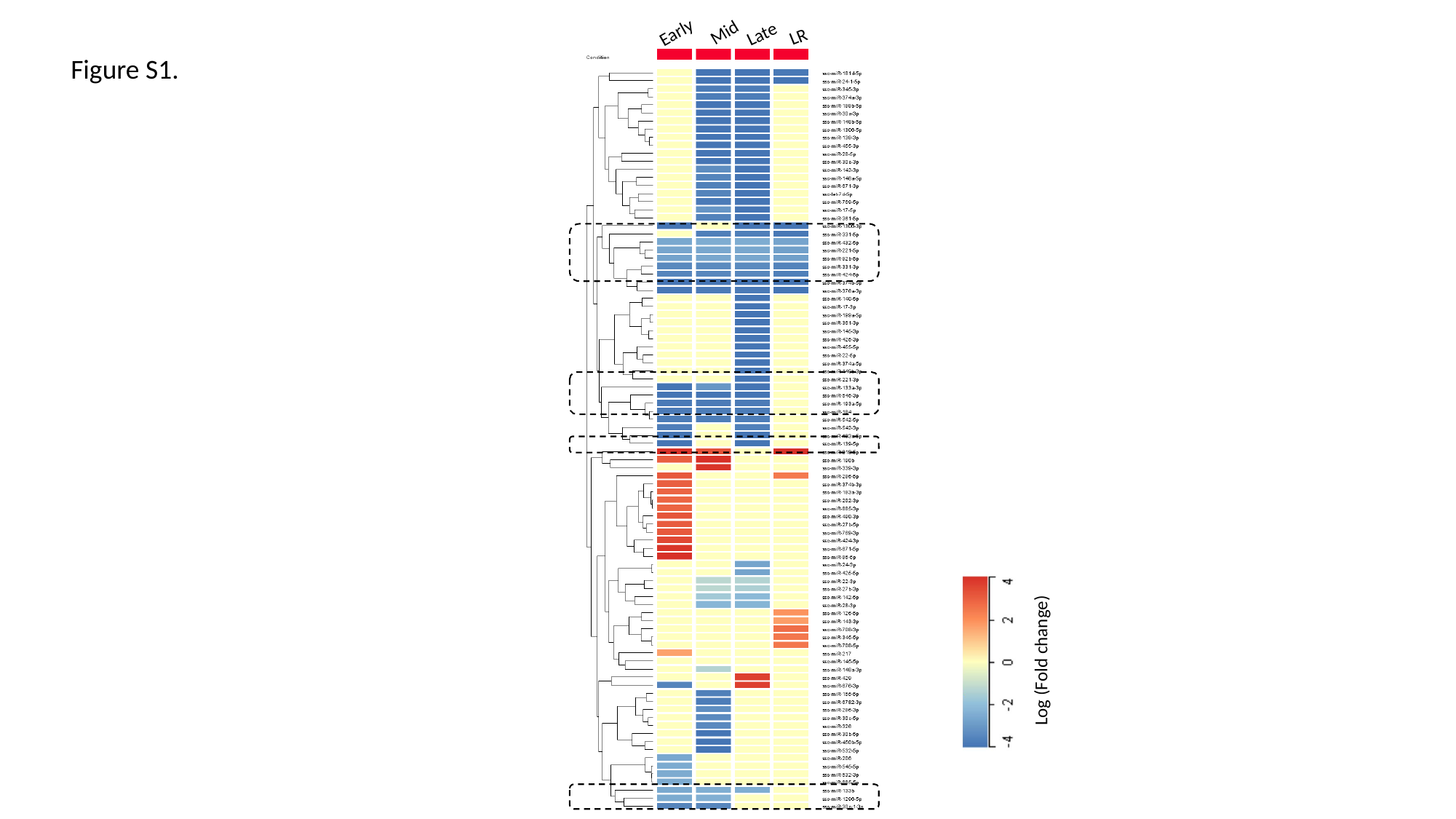

Early
Mid
Late
LR
Figure S1.
Log (Fold change)

## Slide 2
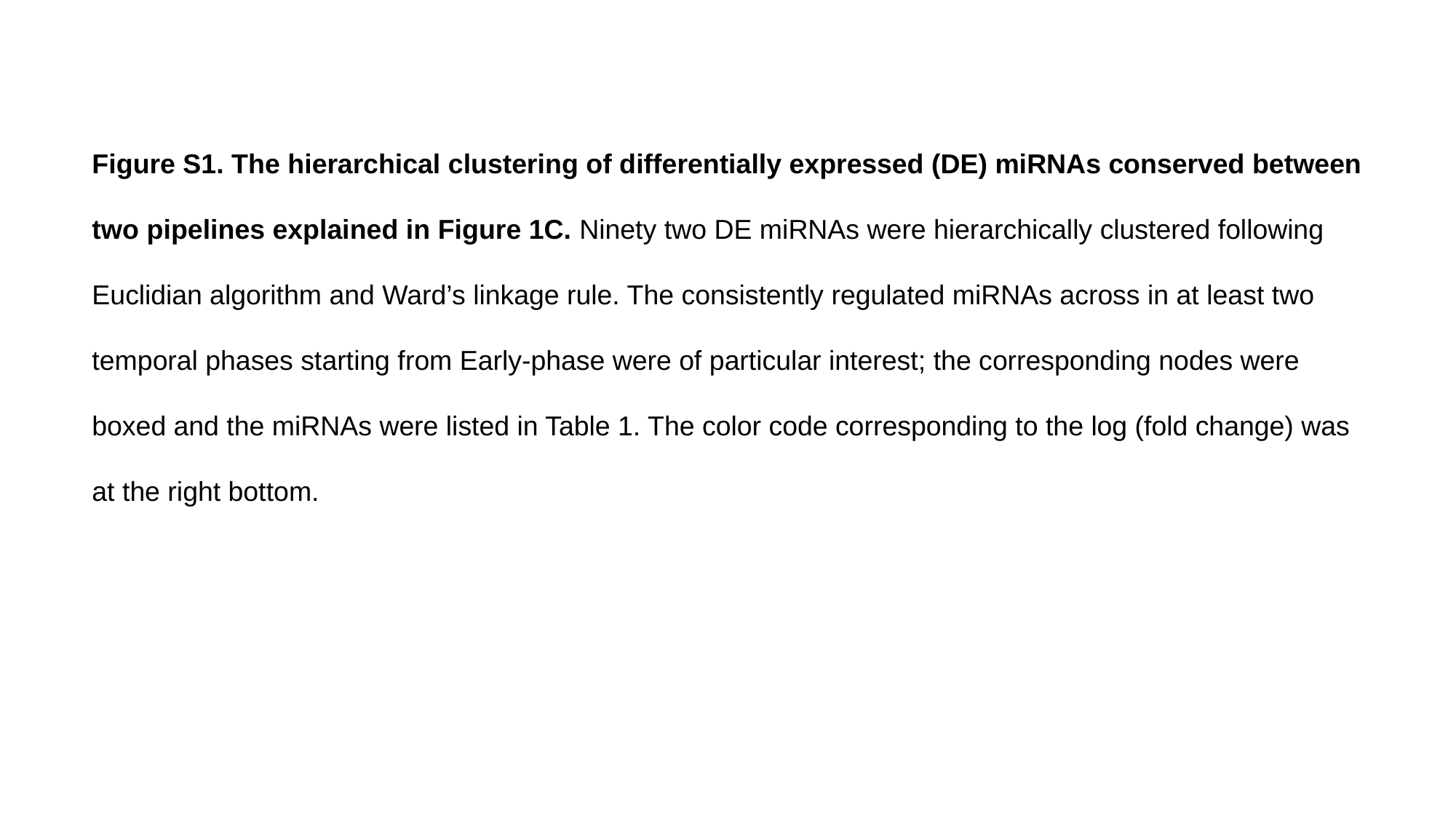

Figure S1. The hierarchical clustering of differentially expressed (DE) miRNAs conserved between two pipelines explained in Figure 1C. Ninety two DE miRNAs were hierarchically clustered following Euclidian algorithm and Ward’s linkage rule. The consistently regulated miRNAs across in at least two temporal phases starting from Early-phase were of particular interest; the corresponding nodes were boxed and the miRNAs were listed in Table 1. The color code corresponding to the log (fold change) was at the right bottom.

## Slide 3
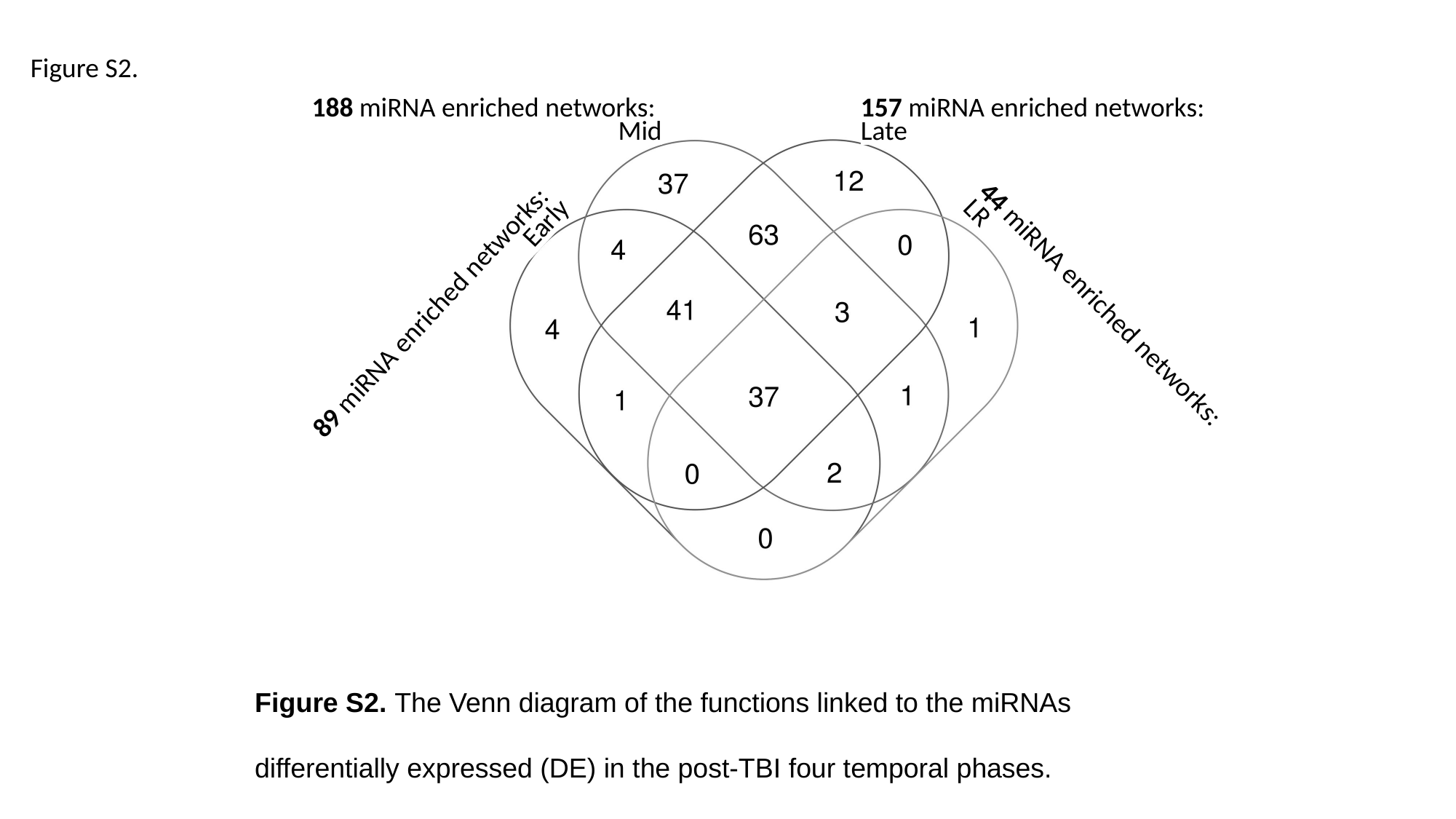

Figure S2.
188 miRNA enriched networks:
Mid
157 miRNA enriched networks:
Late
44 miRNA enriched networks:
LR
89 miRNA enriched networks:
Early
Figure S2. The Venn diagram of the functions linked to the miRNAs differentially expressed (DE) in the post-TBI four temporal phases.

## Slide 4
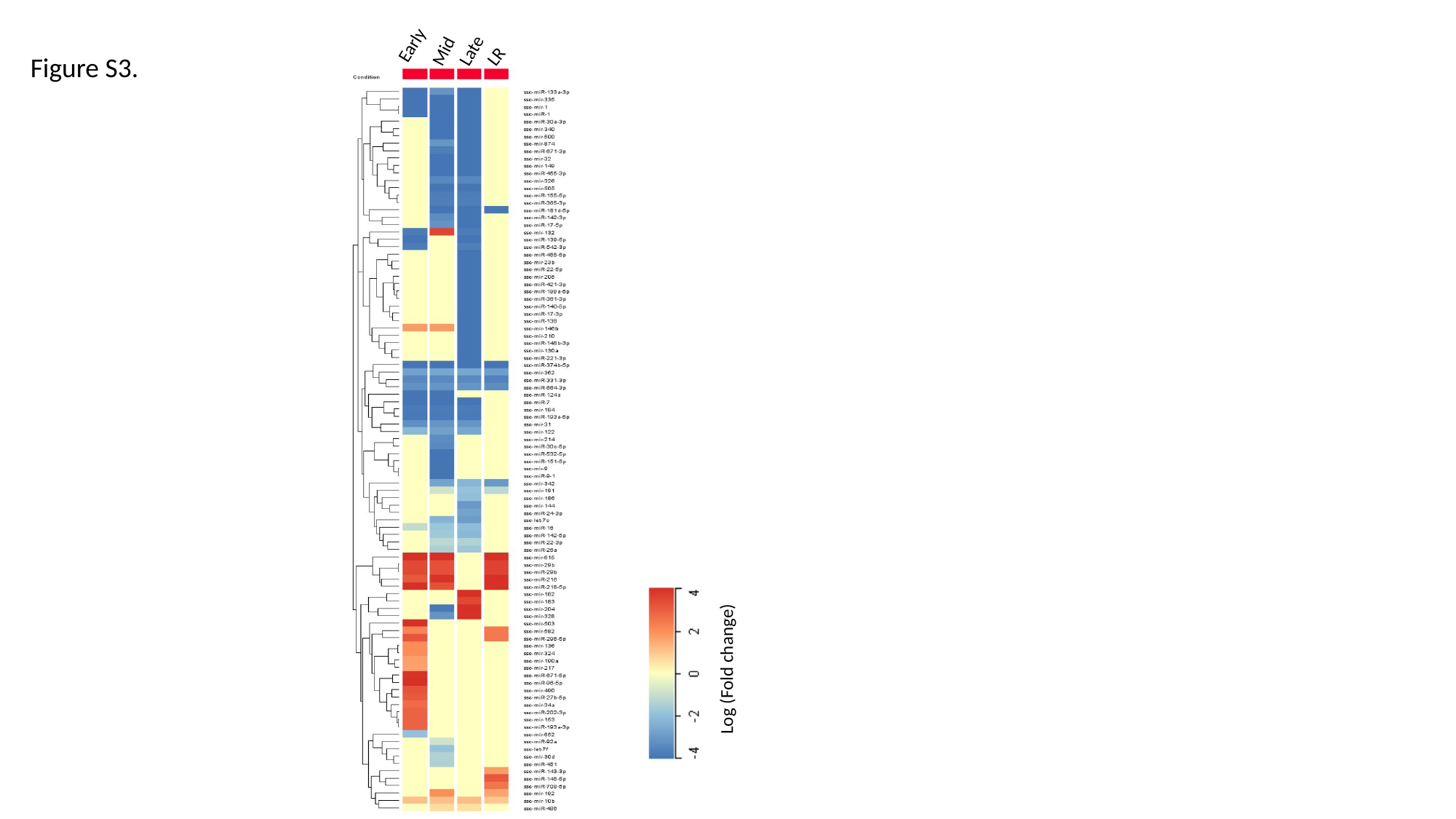

Early
Late
Mid
Figure S3.
LR
Log (Fold change)

## Slide 5
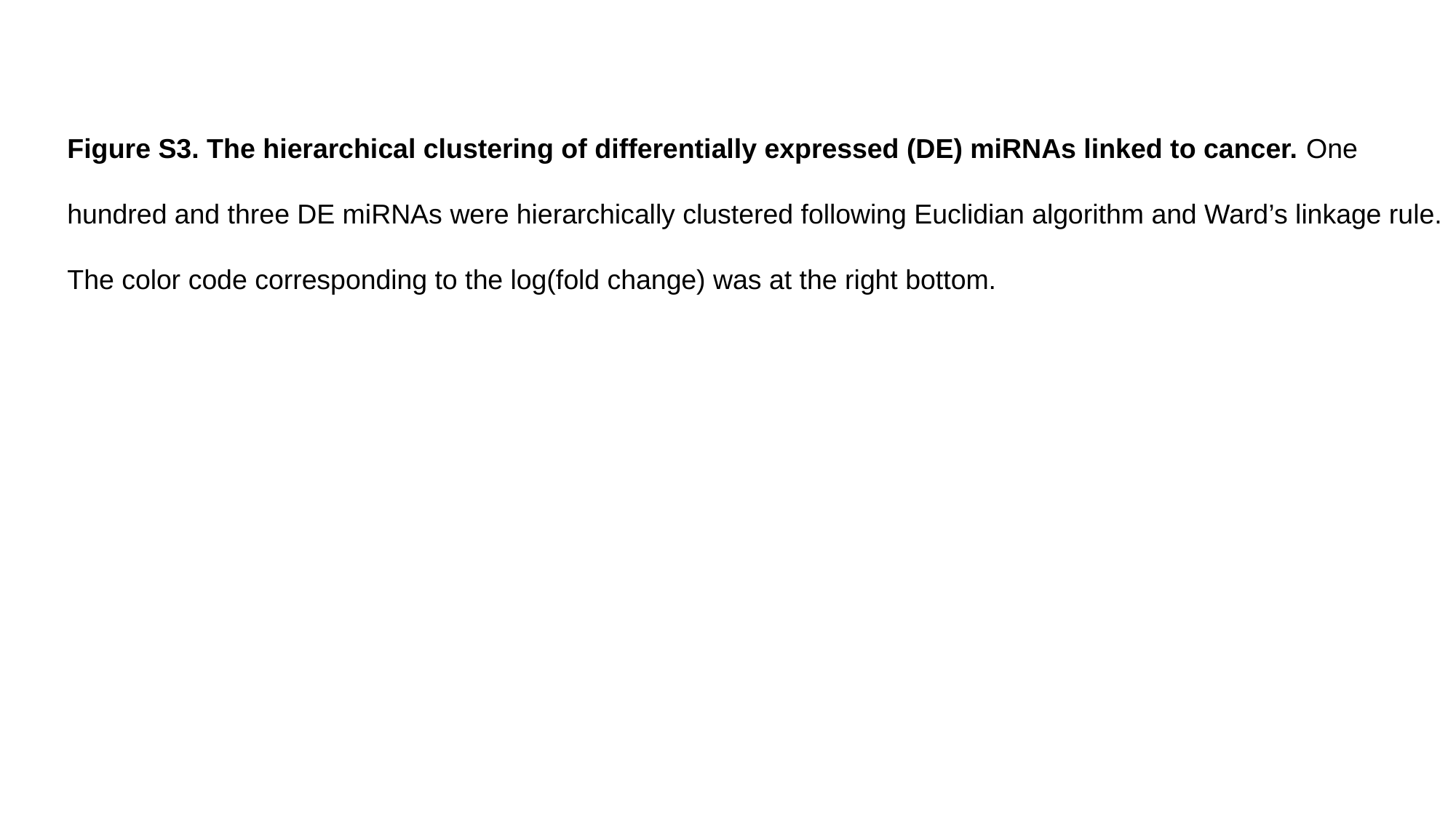

Figure S3. The hierarchical clustering of differentially expressed (DE) miRNAs linked to cancer. One hundred and three DE miRNAs were hierarchically clustered following Euclidian algorithm and Ward’s linkage rule. The color code corresponding to the log(fold change) was at the right bottom.
